# Design, Development, and Testing of BEST4Baby, an mHealth Technology to Support Exclusive Breastfeeding in India: Pilot Study

**DOI:** 10.2196/32795

**Published:** 2022-09-08

**Authors:** Tony Ma, Katie Chang, Amal Alyusuf, Elina Bajracharya, Yukiko Washio, Patricia J Kelly, Roopa M Bellad, Niranjana S Mahantashetti, Umesh Charantimath, Vanessa L Short, Parth Lalakia, Frances Jaeger, Shivaprasad Goudar, Richard Derman

**Affiliations:** 1 Benten Technologies, Inc Manassas, VA United States; 2 Substance Use, Gender and Applied Research RTI International Research Triangle Park, NC United States; 3 Thomas Jefferson University Philadelphia, PA United States; 4 KLE Academy of Higher Education and Research Jawaharlal Nehru Medical College Belagavi, Karnataka India

**Keywords:** mobile health, mHealth, peer counselors, breastfeeding, rural, usability, low- and middle-income countries, agile, task shifting, user-centered design, mobile phone

## Abstract

**Background:**

Exclusive breastfeeding (EBF) at 6 months of age in most low- and middle-income countries, including India, is surprisingly low. There is a relative lack of mobile health apps that specifically focus on leveraging the use of peer counselors (PCs) to support mothers as a means of increasing EBF practices in low- and middle-income countries.

**Objective:**

This study aimed to design, develop, and test the usability of Breastfeeding Education Support Tool for Baby (BEST4Baby), a mobile health app specifically designed to support PCs in providing in-home breastfeeding counseling support to mothers in rural India on optimal breastfeeding practices.

**Methods:**

A user-centered design process with an agile development methodology was used. The approach involved stakeholders and mothers who were trained to serve as PCs to guide BEST4Baby’s design and development, including the app’s content and features. PCs were engaged through focus groups with interactive wireframes. During the 24-month pilot study period, we conducted a feasibility test of the BEST4Baby app with 22 PCs who supported home visits with mothers residing in rural India. The intervention protocol required PCs to provide education and follow mothers using the BEST4Baby app, with 9 scheduled home visits from the late prenatal stage to 6 months post partum. BEST4Baby’s usability from the PCs’ perspective was assessed using the translated System Usability Scale (SUS).

**Results:**

The findings of this study align with best practices in user-centered design (ie, understanding user experience, including context with iterative design with stakeholders) to address EBF barriers. This led to the cultural tailoring and contextual alignment of an evidence-based World Health Organization breastfeeding program with an iterative design and agile development of the BEST4Baby app. A total of 22 PCs tested and rated the BEST4Baby app as highly usable, with a mean SUS score of 85.3 (SD 9.1), placing it over the 95th percentile for SUS scores. The approach translated into a highly usable BEST4Baby app for use by PCs in breastfeeding counseling, which also statistically increased EBF practices.

**Conclusions:**

The findings suggest that BEST4Baby was highly usable and accepted by mothers serving as PCs to support other mothers in their EBF practices and led to positive outcomes in the intervention group’s EBF rates. The pilot study demonstrated that using the specially designed BEST4Baby app was an important support tool for mothers to serve as PCs during the 9 home visits.

**Trial Registration:**

Clinicaltrials.gov NCT03533725; https://clinicaltrials.gov/ct2/show/NCT03533725

## Introduction

### Exclusive Breastfeeding

Exclusive breastfeeding (EBF), defined as *the practice of giving an infant only breast milk for the first 6 months of life*, has tremendous benefits for the mother-infant dyad. It protects the infant from infections, sudden infant death syndrome, dental malocclusions, and the development of obesity and diabetes later in life [1-4]. EBF offers protection for the mother from breast cancer, ovarian cancer, and postpartum depression and improves the birth spacing interval [1,3,4]. Of great significance, EBF has been shown to be most effective in reducing overall infant mortality [5], which could prevent an estimated 823,000 child deaths [2-4] and an annual economic loss of approximately US $302 billion. EBF practices can significantly contribute to improved outcomes in under resourced environments [6].

Low- and middle-income countries (LMICs) have the highest infant mortality rates (IMRs), and approximately 63% of infants aged <6 months are not exclusively breastfed [3]. This is especially important in India, which has reported an IMR of 30 per 1000 live births. India’s IMR exceeds that of several other LMICs in Asia, such as Uzbekistan (19 per 1000 live births), Bangladesh (25 per 1000 births), and Egypt (18 per 1000 live births). Despite the Indian government’s longstanding efforts to increase breastfeeding rates, EBF remains low. The National Family Health Survey 2015 to 2016 estimated that only 54.9% of infants aged <6 months are exclusively breastfed in India [7]. Furthermore, EBF rates are not homogenous in India and vary by region [8], with rates in Southern India reported to be 43.7% from infancy to the age of 5 months [8].

### Socioecological Factors of EBF

Multiple socioecological factors affect EBF rates in India. The social determinants of health include a mother’s access to health care and health information, education, income, and area of residence [9], all of which affect the rates of EBF. The mother’s lack of knowledge on the importance of EBF to overall health, inappropriate breastfeeding techniques, and late initiation of breastfeeding are barriers at the individual level [10-13]. At the societal level, discarding colostrum, prelacteal feeding, lack of family support, lack of the mother’s autonomy in decision-making (eg, mother-in-law’s influence on breastfeeding practice), and poor counseling regarding key aspects of EBF are cited as significant barriers [9,12,13]. In South Asia, programs that used repeated exposures for counseling and education on breastfeeding practices during pregnancy and in the early postpartum period were more likely to be effective in improving breastfeeding rates. Short programs that contained irregular exposures, poor timing, and inadequate coverage of the target population were less likely to affect breastfeeding rates.

### Breastfeeding Support System, the Role of Community Health Workers, and the Importance of Task-Shifting Breastfeeding Counseling In India

Task shifting is a delegation process in which tasks are moved from a highly specialized workforce to less specialized health workers, where appropriate. The Indian government has implemented and deployed different types of paid or incentivized community health workers (CHWs) as part of India’s health care delivery system to task shift various health care activities [14,15]. Among the 3 cadres of CHWs implemented, auxiliary nurse midwives (ANMs) are based at a subcenter and visit villages and provide care at the subcenter. ANMs are supported by Anganwadi workers, who work solely in their villages and focus on providing food supplements to young children, adolescent girls, and lactating women. Finally, Accredited Social Health Activists (ASHAs) are the largest cadres of CHWs. They have been deployed to supplement the work of ANMs and Anganwadi workers [15]. ASHAs provide health promotion, specifically regarding nutrition, sanitation and hygiene, preparedness for birth and safe delivery, immunization, breastfeeding, complementary feeding, and prevention of common infections. Although they provide a valuable contribution to supporting and promoting maternal and child health in their communities, ASHAs often feel rushed, tired, overworked, and underpaid [16].

Studies using CHWs have generally shown significant improvements in general maternal-infant care practices (eg, skin-to-skin care) and some areas of breastfeeding support, including initiation and complementary feeding [17,18]. Furthermore, establishing a network of CHWs who can educate, support, and make necessary referrals is linked to increasing EBF [19]. Although paid or incentivized and trained CHWs have been used for breastfeeding interventions in India, the use of mothers as unpaid peer counselors (PCs) for breastfeeding has not been explored.

### Technology-Based Support for CHWs

Mobile technology for health interventions presents a strong opportunity for improving breastfeeding practices compared with usual care, including the cost-effectiveness of using CHWs such as ASHAs. Mobile health (mHealth) apps that rely on wireless access to the internet are common in India, where broad access is available, including in rural areas. Several mHealth interventions have been tested in rural areas in India to improve maternal-child health outcomes, including breastfeeding [17,18,20]. These mHealth intervention studies using CHWs [17], such as ASHAs [18], have reported improvements, including increases in job confidence [17], coordination [17], coverage [18], and quality of services in hard-to-reach areas [18]. Despite established evidence of the effectiveness of using CHWs to provide community-based counseling and education to improve EBF rates [9,21-25], the use of mHealth with task shifting to unpaid mothers as PCs in India has yet to be explored. Brief training with mothers with prior breastfeeding experiences to serve as PCs, using mHealth technology as suitable support tools, within rural Indian communities can further expand the types of available CHWs. They would further address the need for community-based peer support to promote breastfeeding in rural India [26]. Thus, Breastfeeding Education Support Tool for Baby (BEST4Baby) is a suitable medium for training and supporting unpaid PCs in providing in-home breastfeeding counseling support to mothers in rural India.

## Methods

### Overview

This paper describes the design, development (ie, content and technical), and testing of the mHealth app called the *BEST4Baby* to support a community-based task-shifting intervention that uses trained mothers as PCs to provide counseling and education to mothers to achieve an improved EBF rate in the Belagavi district of Karnataka, India. The investigators included partners at Thomas Jefferson University (TJU; Philadelphia, Pennsylvania, United States) and investigators at Jawaharlal Nehru Medical College of Karnataka Lingayat Education, Academy of Higher Education and Research (Belagavi, India), and Benten Technologies (Benten; Manassas, Virginia, United States).

**Figure 1:**
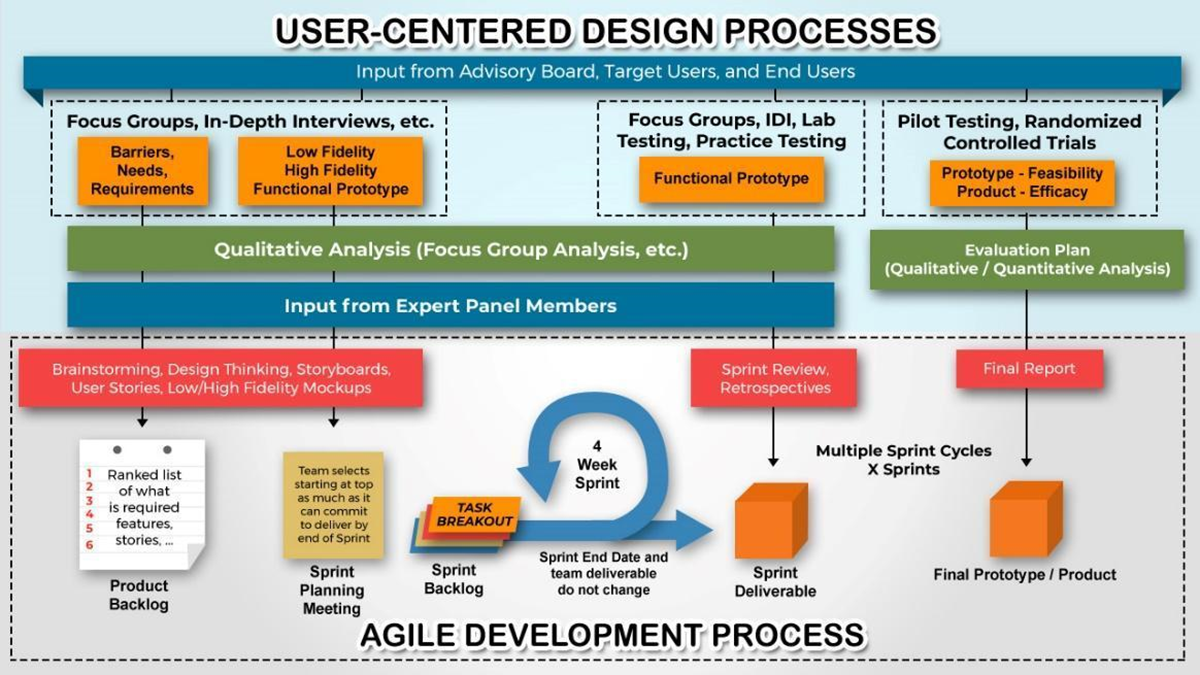
The Breastfeeding Education Support Tool for Baby app design and development process. IDI: in-depth interview.

The team leveraged a user-centered design (UCD) approach to design and develop the initial version of the BEST4Baby mHealth app. This approach was used to better understand the users, counseling tasks, and the environment by involving PCs and key stakeholders throughout the design process to create a positive user experience [27]. This process ensured that the BEST4Baby counseling program and the training materials, mobile app, and job-aid tools were acceptable and feasible in advance of pilot testing. The team also leveraged an agile development methodology for content and technology development. The agile development methodology emphasizes iterative development and integrates feedback from all key stakeholders in the development process to refine the BEST4Baby app over time, including the content and app [28]. All design and development processes were initially conducted in English to facilitate communication among all research team members during the study. Meetings were conducted web-based via UberConference and Zoom. Figure 1 illustrates the UCD process implemented alongside the agile development of the BEST4Baby mHealth app. The mHealth app was then pilot tested for usability with PCs to deliver effective EBF counseling to mothers at home.

### BEST4Baby App Design

#### Overview

Formative qualitative research was conducted with a breastfeeding advisory panel and mothers with and without breastfeeding experience [19]. The members of the breastfeeding advisory panel included local clinicians and academics incorporating the medical disciplines of obstetrics and pediatrics, representatives of advocacy groups, a Karnataka State Ministry of Health official, the Reproductive and Child Health Officer, and the District Health Officer from Belgaum, India. The results of the qualitative research and analyses helped the team to create an initial product backlog for the BEST4Baby mHealth app. Microsoft PowerPoint was used as a design tool to iteratively create mock-ups for the BEST4Baby mHealth app. The design for the initial mock-ups was based on information derived from formative qualitative research and informed the app’s structure of number, timing, spacing of visits, and specific content that would be reviewed during the visits and incorporated cultural practices during various stages of breastfeeding. Initially, the BEST4Baby app focused on addressing the importance of identifying salient barriers to breastfeeding.

#### Design Iterations

The design versions for the BEST4Baby app included the (1) initial mock-up design, (2) revised mock-up design with integrated educational content, and (3) final interactive wireframes with content. The stakeholders were engaged in the feedback of the design mock-ups to ensure proper flow and integration with BEST4Baby PC training. Changes in the design included simplifying navigation options, removing one prenatal visit, and adding the potential to make unscheduled visits. The final design integrated all the educational content and was reviewed and approved by our research collaborators in India.

#### Visit Session Design

The BEST4Baby app design centered on the following concepts: (1) time (relatively short sessions), (2) content (small, bite-size units of information to be covered in each session), (3) curriculum (personalized content for each session as part of a curriculum), (4) form (all sessions presented in the same, consistent structure and format, as well as incorporating multimedia when appropriate), and (5) flexibility (different times and locations for each delivery) [29]. Mothers who served as PCs were the primary users of the app. After brief training, the PCs could use the mHealth app to master complex breastfeeding counseling topics by using many short, guided visit sessions with a built-in refresher training module for use in the field. Each visit session was designed to dynamically present content for PCs to share and counsel mothers based on their responses at each visit. The app was designed to provide just-in-time information without overloading mothers with too much information. With scheduled visits by PCs at various stages (before and after delivery), mothers were only presented with stage-appropriate content for that period. For example, visit 2 was 32 to 36 weeks ante partum and provided information on the importance of colostrum; visit 3, which occurs within 1 to 3 days after delivery, focuses on issues related to delivery, such as baby delivery questions (baby’s weight, birthing hospital, and delivery method), feeding questions, assessment of baby and mother’s health, and others. The app was also designed with assessments for PCs conducted at the beginning of each visit to identify potential problems. On the basis of the assessment results, PC counseling content was dynamically shown to address specific solutions for mothers.

### BEST4Baby Content Development

#### Overview

In parallel with the app design, the educational content for the BEST4Baby mHealth intervention was developed with experts in the field. The educational content leveraged the formative research with mothers. It noted content that was successful and unsuccessful in promoting EBF [19], results from a prior study, and input from PCs to finalize the design. The educational content was based on a modified, culturally adapted World Health Organization breastfeeding counseling course. The content was created for training PCs and, as appropriate, for use during in-home and hospital visits to improve the mother’s knowledge regarding EBF and optimal feeding practices [30]. The content included videos comprising brief but common experiences prevalent in breastfeeding practices. The video content was tested for compatibility with Android and iOS devices.

#### Behavioral Theories

The design of the BEST4Baby educational content leveraged the Social Cognitive Theory (SCT) and the Theory of Planned Behavior (TPB) [31]. The SCT’s construct of *self-efficacy* was used to develop educational content using videos of other Indian mothers who were successful in various breastfeeding techniques. The SCT’s constructs of *outcomes expectancy* and *observational learning* were incorporated into design components that addressed sociostructural factors that facilitate or hinder pursued behavior, such as the mother or mother-in-law’s influence on breastfeeding, prelacteal feeding, and complementary feeding. The TPB’s construct of *behavioral intentions* was incorporated into the BEST4Baby app design. The TPB constructs of *attitude* (eg, assessing initial and subsequent attitudes), *subjective norms* (eg, involving key individuals such as mothers-in-law), and *perceived behavioral controls* (eg, reinforcing a mother’s self-efficacy using an observational assessment, personalized counseling to their breastfeeding challenges, and videos to perform the behavior) were incorporated into the step-by-step counseling guide [31,32].

#### Content Integration With App

The educational content was integrated into the BEST4Baby app design as each component of the content was completed. After incorporating this design and content into an interactive wireframe, a focus group session was conducted with 7 PCs to demonstrate the mHealth app and obtain feedback. After the focus group, we administered the System Usability Scale (SUS) survey to evaluate the initial usability of the BEST4Baby app design, obtain final feedback on the design, and incorporate feedback before completing the technological development of the prototype. The SUS is a reliable scale widely used and validated to measure system usability, independent of the type of technology [33]. SUS has also been used in various languages and cultures, including in India [34,35]. It has become an industry standard, with reference in >600 publications [36].

### BEST4Baby Technological Development

The Benten development team consulted TJU off site and the Indian team as product owners during the agile development. In addition, lessons learned from a prior mHealth intervention, called Community Level Interventions for pre-eclampsia [37] in India, provided knowledge on potential infrastructure challenges for the use of mobile technology in rural India.

#### Overall Architecture

The mHealth app was developed with cross-platform mobile technologies using MongoDB, Express, ReactJS, and NodeJS, which is a technology stack available for any Android or iOS device. The initial architecture included web-based and offline capabilities to address wireless coverage issues. The team also developed security features, such as strong password protection for authentication, access control, and secure transport of information via the Secure Sockets Layer, to ensure that the mHealth app conformed to India’s regulations on the privacy and security of patient data. The prototype was also designed to capture data regarding app use to assist in the evaluation of the app. The content leveraged XML to encode the content and allowed for a dynamic, personalized presentation of breastfeeding counseling information based on the data captured for each mother during their visit. At the initial visit, detailed data of app use were captured, including GPS data, time spent by PCs in each content area, and individual responses from mothers during each counseling session.

#### App Features

**Figure 2:**
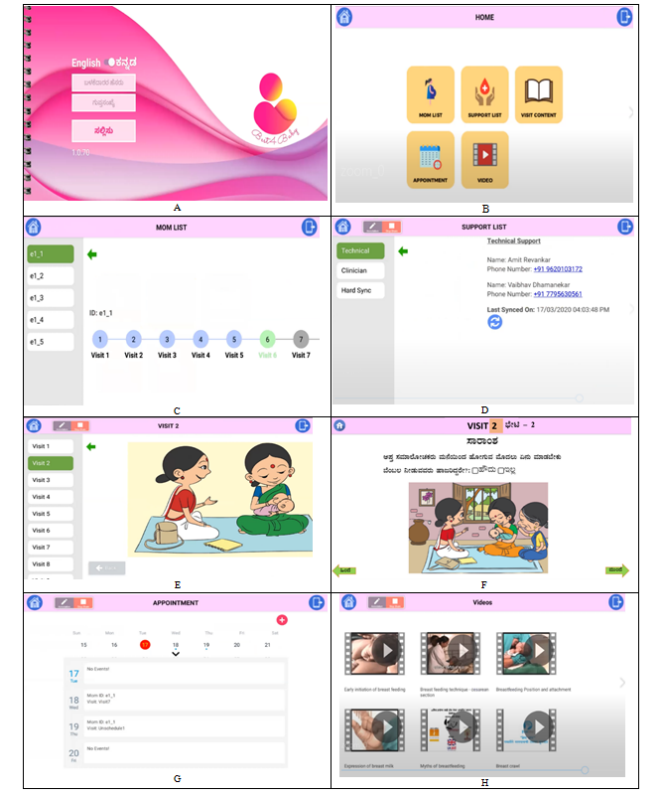
Screenshots of the Breastfeeding Education Support Tool for Baby mobile health app. A. Login screen of the BEST4Baby app B. BEST4Baby Homepage C. App displaying visit numbers and assigned moms D. App displaying support list for technical and clinical support E. App displaying visit content (In English) F. App displaying visit content (In Kannada) G. App displaying appointment calendar H. App displaying educational videos on breastfeeding.

The team created the BEST4Baby app to provide a systematic step-by-step guide [26,27] to control the quality of PC counseling at each visit and promote optimal breastfeeding practices in mothers. BEST4Baby’s unique features included (1) a step-by-step guide for each visit on proper breastfeeding counseling and education, (2) systematic breastfeeding assessments for mothers during the initial prenatal and postpartum period to shape beliefs and troubleshoot breastfeeding challenges, (3) time-sequenced prenatal and postpartum period education to deliver appropriate just-in-time breastfeeding information, and (4) personalized communication to address specific breastfeeding challenges of lactating mothers during the postpartum period. The PCs accessed the BEST4Baby app using a simplified log-in process. Once authenticated, a PC could view the educational content and conduct in-home visits in English or the local language (Figure 2). The BEST4Baby home page provides access to functionality, such as the (1) Mom List, which allows PCs to add to and track visits for each designated mother, including completed and pending visits; (2) Support List, which allows PCs to communicate with clinicians and technical support as needed; (3) Visit Content, which provides step-by-step guided counseling incorporated within the educational content, including multimedia and video clips, organized into 9 sessions corresponding to the visits by PCs to mothers (2 antenatal and 7 post partum); (4) Appointments, which allows PCs to view upcoming visits, schedule new appointments, and add them to the calendar; (5) Video, which provides PCs with a breastfeeding content library for viewing directly whenever needed during the visit.

#### Integration and Testing Preparation

After development, the Indian team translated the final content into the local language, Kannada. The translation was provided in Unicode for incorporation into the BEST4Baby app. The app was designed to display the content and interface components in English and Kannada. In preparation for the pilot study, the Indian team researched possible low-cost Android devices, which constitutes >90% of the market in India [38]. Different Android tablet devices were considered. A Samsung Android tablet was chosen as it was an affordable, common, easily accessible, and portable device that could fit in the BEST4Baby kit for PCs to carry to the pilot testing site. The kit had the following items: (1) a life-size newborn doll to demonstrate positioning, (2) a skin-colored sock to prepare a breast model for demonstrating proper latching, (3) a digital scale for weighing and assessing the growth of the infant, (4) a nipple plunger to mitigate the problem of an inverted nipple, and (5) a Samsung Android tablet with wireless and GPS capability preloaded with the BEST4Baby app and secured to allow for its sole use with the app. A total of 25 Samsung tablet devices were purchased for use by the PCs for the study implementation. Final testing was performed to ensure compatibility of the final BEST4Baby software with the device selected for the PCs.

### Usability Testing

#### Study Participants

A total of 56 potential PCs were identified by staff from 5 local primary health centers in the Belagavi health district, facilitated through announcements and word of mouth [26]. Out of 56 PCs, 25 (45%) were selected for usability testing of the platform based on the inclusion criteria of (1) residing in the local community; (2) having breastfed within the past 5 years; (3) having at least 10 years of formal education; (4) having an available mobile phone; (5) being familiar with operating an Android phone; and (6) being able to read, write, and communicate in the local language.

#### Study Design

Usability refers to “the quality of a user’s experience when interacting with products or systems, including websites, software, devices, or applications” [39]. A sample size of 20 PCs was sufficient to achieve 80% power to detect a difference of 6.0 between the actual mean of 74.0 and the null-hypothesized mean of 68.0. The International Organization of Standardization 9241-11 considers usability to be a measure of the system’s technical effectiveness, efficiency, and satisfaction from the perspective of user experiences [40]. BEST4Baby usability was tested in a real-life setting within 6 clusters of the Global Research Network area located in Belagavi district (Karnataka, India). The pilot study involved pretesting for usability with 25 PCs and posttesting for usability with 22 PCs (88%; n=3, 12% dropped out from the study) recruited from the study areas.

#### Study Procedure

Following recruitment, all 25 PCs attended a 3-day training session, which included content on breastfeeding knowledge and skills, counseling techniques, and instruction on the BEST4Baby mHealth app. Each PC received an ID badge and a branded BEST4Baby mobile device containing the BEST4Baby mobile app. During the posttraining time point, each PC was assigned to 5 mothers in the intervention group, who resided in their community. PCs used the BEST4Baby app during home visits to mothers following a 9-visit schedule at >28 to 32 weeks ante partum, 32 to 36 weeks ante partum, postpartum days 1 to 3, postpartum day 7, postpartum day 15, postpartum 1 month, postpartum 2 months postpartum 4 months, and postpartum 6 months. As the PCs went to the visit sessions with the mothers, they manually entered and collected data using the BEST4Baby app on the assigned Samsung tablet. Data from the Samsung tablet automatically synchronizes to a server if the internet is available. The BEST4Baby app was also designed with the capability of an offline mode feature, which means that the educational content for the visits was stored on the Samsung tablet, and data could be collected and stored on the tablet without the need to access the internet or wireless service to address potential wireless coverage issues in a rural area. The offline mode allowed the data to be synchronized later to the server whenever the app could detect internet service availability. In addition, the app also had a feature to enable PCs to synchronize the data manually. At the end of the training, 25 PCs were asked to complete a SUS survey to assess the usability and acceptability of the BEST4Baby app. The SUS questionnaire was translated into the local language. The results from the SUS score were found to be highly usable, with an average score of 87.5 (SD 8.2; range 72.5-100).

#### Instruments

BEST4Baby’s usability was assessed using the SUS [33,36], a widely used validated scale that can be used with a variety of technologies and provides a single usability score [36]. The survey comprises a 10-item Likert scale for respondents (with 5-point anchors from strongly agree to strongly disagree). Scores for each item in the SUS survey are converted to a number, added together, and then multiplied by 2.5 to create a single SUS score between 0 and 100. A SUS score >68 is considered above average and supports acceptability for use [36]. Data regarding app use were automatically captured from the app and obtained from the server to assess user engagement with the BEST4Baby app.

#### Usability Data Analysis

Data were collected from the surveys, deidentified, and entered into a Microsoft Excel spreadsheet. After verifying the data, baseline demographics were assessed using descriptive statistics. Individual SUS scores were calculated for each participant, and a mean SUS score with SD was provided for postpilot survey results. App use data were obtained from the server after the pilot and analyzed using descriptive statistics.

### Ethics Approval

Institutional review board approval was obtained from Karnataka Lingayat Education University (IRB registration number: 00008025), Jawaharlal Nehru Medical College in India, and TJU in the United States.

## Results

### Participant Characteristics

A total of 25 participants were recruited as PCs, of whom 3 (12%) did not complete the study, resulting in 22 (88%) participants who completed both the study and posttesting usability survey. The usability survey was translated into the local language before it was administered. The participants were all female, with a mean age of 30.18 (SD 4.43) years [26]. Half of the participants reported having at least 8 to 10 years of education, with the remainder having 11 to 16 years of education; all had prior experience of using a smartphone for an average of 14.95 months (Table 1).

**Table 1:** Demographics (N=22).

Characteristics		Values
Age (years), mean (SD)		30.18 (4.43)
**Age (years), n (%)**		
	21-25	3 (14)
	26-30	11 (50)
	31 35	6 (27)
	36-40	2 (9)
**Years of education, n (%)**		
	8-10	11 (50)
	11-16	11 (50)
Smartphone experience (months), mean (SD)		14.95 (21.33)

### App Use Data

A total of 22 PCs completed the 9-visit schedule with mothers, with each PC serving 4 to 5 mothers. PCs also had the option of adding additional unscheduled visits (a feature that was optional and accessible in the app). Unscheduled visits were designed for the PCs to provide additional breastfeeding support if needed. The time that the PCs spent on the app with the mothers during each visit ranged from 6.6 to 39.6 minutes. The longest period was during the first visit, whereas the shortest was during the unscheduled visits. The average time spent by PCs on visits 1 to 5 was 29.1 minutes, whereas the average time for later visits (eg, visits 6-9) was 12.7 minutes (Table 2). After the first 6 visits, PCs spent an average of <12 minutes on the last 3 visits (2-, 4-, and 6-month visits post partum). Unscheduled visits (ie, visits that were not originally planned) also had the shortest duration (<7 minutes).

During each visit, PCs could revisit the educational content with mothers, which was provided previously (another optional feature of the app) based on individual needs. The most frequently revisited educational content topics included the importance of colostrum feeding, position and attachment, and proper burping after feeding. The content related to the importance of colostrum feeding, the many advantages of breastfeeding, and the dangers of artificial and prelacteal feeding were also revisited during visit 3 (Table 3). Content related to the demonstration of position and attachment, expression of breast milk, increased secretion of breast milk, and proper burping after a feed was revisited during visits 4 and 5 (Table 3).

**Table 2:** Visit duration.

Visit name	Targeted visit time points	Average duration per visit	Duration per screen, mean (SD)
Visit 1	28-32 weeks ante partum	39.6	1.41 (1.69)
Visit 2	32-36 weeks ante partum	24.0	1.09 (1.50)
Visit 3	Postpartum days 1-3	28.1	0.76 (0.82)
Visit 4	Postpartum day 7	28.5	0.81 (0.95)
Visit 5	Postpartum day 15	25.4	0.73 (0.89)
Visit 6	Postpartum 1 month	16.6	0.57 (0.50)
Visit 7	Postpartum 2 months	11.2	0.56 (0.37)
Visit 8	Postpartum 4 months	11.0	0.55 (0.40)
Visit 9	Postpartum 6 months	11.9	0.56 (0.40)
Unscheduled visits	As needed between visits	6.6	0.28 (0.37)

**Table 3:** Topics revisited and time frame of when it occurred.

Content topic	Revisits, N	When it occurred
Flat or inverted nipples	3	Unscheduled visits
Breast engorgement	4	Unscheduled visits
Sore nipples	2	Unscheduled visits
Importance of colostrum feeding	95	Visit 3
Advantages of breastfeeding	32	Visit 3
Dangers of artificial feeding	23	Visit 3
Dangers of prelacteal feeding	24	Visit 3
Demonstrate position and attachment	111	Visits 4 and 5
Expression of breast milk	66	Visits 4 and 5
How to increase secretion of breast milk	58	Visits 4 and 5
Proper burping after a feed	116	Visits 4 and 5

### Usability Analysis

The SUS scores for PCs ranged from 60 to 97.5, with a mean SUS score of 85.3 (SD 9.1). The mean SUS score, which was above the *acceptable* score [33], was considered above average by industry standards, indicating high usability. The average SUS score achieved by PCs places the BEST4Baby mHealth app above the 95th percentile in terms of usability [41]. All 22 participants strongly agreed with the following statements: “I would use this app frequently,” “I found the app easy to use,” and “It was easy to use the app during training.” In addition, 91% (20/22) of PCs strongly agreed with the statements that “The app features are well integrated” and “I can easily learn to use this app.” Most PCs strongly disagreed with the statements that “There was inconsistency in the app” and “I found the app too complex.” However, half of the PCs strongly agreed with “I needed to learn many things before I could use the app” (Table 4).

**Table 4:** Individual SUS^a^ item scores (N=22).

SUS item	Statement (rank your impression from 1 to 5; 1=strongly disagree and 5=strongly agree)	Values, mean (SD)	5=strongly agree, n (%)	1=strongly disagree, n (%)
1	I would use this app frequently	5 (0)	22 (100)	0 (0)
2	I found the app too complex	1.6 (0.8)	0 (0)	13 (59)
3	I found the app easy to use	5 (0)	22 (100)	0 (0)
4	I need a technical person to use this app	2.3 (0.9)	0 (0)	4 (18)
5	The app features are well integrated	4.9 (0.3)	20 (91)	0 (0)
6	There was inconsistency in the app	1.4 (0.9)	1 (5)	17 (77)
7	I can easily learn to use this app	4.6 (1.2)	20 (91)	2 (9)
8	I found the app cumbersome to use	1.5 (1.1)	1 (5)	16 (73)
9	It was easy to use the app during training	5 (0)	22 (100)	0 (0)
10	I needed to learn many things before I could use the app	3.6 (1.6)	11 (50)	4 (18)

^a^SUS: System Usability Scale.

## Discussion

### Principal Findings

We piloted BEST4Baby with 22 PCs to design, develop, and test the usability of this mHealth app, specifically designed to support PCs in providing in-home breastfeeding counseling to mothers in rural areas. The results of this usability test suggest that BEST4Baby was highly usable and acceptable by PCs in supporting mothers on optimal breastfeeding practices. The BEST4Baby mHealth app was developed following industry best practices and standards for UCD and agile development for use by PCs to support EBF practices in India. Development used a UCD approach to better understand the user experience [27]. The UCD process led to the creation of a unique app design that yielded a high usability rating by the mothers as PCs, resulting in high EBF rates at 6 months.

Based on the findings, the research team developed unique content and app features to support PCs in delivering in-home breastfeeding counseling, including the design of *just-in-time* information and skill-building content for mothers just before they require the information, as well as the use of checklists to guide the assessments, which included dynamic content delivery for each session specific to the mother’s challenges or needs. With only 3 days of training on breastfeeding counseling, including the use of the BEST4Baby app, mothers serving as PCs were able to learn how to use the app to conduct visit sessions.

The BEST4Baby app was pilot tested in ecologically valid settings in the Belagavi district in India and was determined to be highly usable and acceptable by PCs in this study. PCs used the BEST4Baby mHealth app to support a 9-visit intervention. The app’s features, including calendar scheduling, reminders, and dynamically generated content, helped support mothers to serve as PCs to meet breastfeeding counseling visit schedules designed for the intervention. Initial visits lasted longer, whereas later visits were shorter (eg, an average of 29 minutes vs an average of <12 minutes). The most frequently revisited educational topics were related to the importance of colostrum feeding, position and attachment, and proper burping after feeding. In addition, the results related to content topics and when they were discussed were based on the specific needs to address each mother’s challenge during the visits. The findings from the PCs will help inform future modifications of the BEST4Baby software.

A unique aspect of our mHealth app was the step-by-step guide with breastfeeding assessment, which provided dynamic counseling content to support mothers and created an intuitive flow for each visit. A step-by-step guide is a crucial component for activating desired behavior, as applied in evidence-based digital health interventions for mental health and behavior change [42]. Using a step-by-step guide along with a calendar for the scheduling of visits, appointment reminders, breastfeeding assessments, provision of dynamic counseling content, and use of the training module for field practice and refreshers resulted in PCs reporting high usability in supporting mothers to exclusively breastfeed.

Another unique feature of the tested mobile app was the tracking system of the PCs’ adherence to the 9-visit protocol. User engagement can be a challenge when attempting to implement an mHealth intervention [43]. Future research could incorporate components such as gamification to ensure user adherence to the mobile app [44] and protocol content.

### Technical Challenges

During pilot testing, the research team experienced several technical challenges. The first occurred during the deployment of the BEST4Baby app, including data synchronization with the cloud-based server. This was resolved by providing a manual synchronization button. Second, owing to synchronization issues across multiple devices, the research team and PCs could not log into different devices, preventing researchers from monitoring and tracking the progress of each PC in real-time. The team developed a work-around monitoring functionality by creating a weekly export report in Microsoft Excel to overcome this issue. The report provided weekly details so that each PC’s visit history, including completed visits for each mother, could be provided to the research team.

### Strengths

Studies have shown high levels of perceived acceptability of mHealth-supported interventions among CHWs in low-resource settings and LMICs, including in India [45]. A recent meta-analysis of studies conducted in 6 countries suggested that mHealth may be associated with improved maternal breastfeeding attitudes, knowledge, initiation, and EBF duration [46]. However, to the best of our knowledge, BEST4Baby is among the first mHealth apps to test the use of mHealth among unpaid PCs with a limited amount of training in rural India to successfully promote and support optimal breastfeeding practices. To date, no assessment has been made on the use of mHealth to enhance breastfeeding PC programs in India.

### Limitations

This study had certain limitations. First, only feedback from mothers who served as PCs was included in the initial design process, and the input of mothers served by the PCs was not sought. Second, usability and acceptability were collected at the beginning and end of the pilot for the PCs who had breastfeeding experiences within 5 years; however, the perspective on the usability of BEST4Baby from current breastfeeding mothers was not collected. Third, owing to our small sample size of PCs to evaluate feasibility (usability), the small sample size was not powered to evaluate the study’s external validity. Finally, we were also unable to explore the characteristics of PCs who reported below-average SUS scores because of our small sample size.

### Conclusions

Our findings suggest that an mHealth tool such as the BEST4Baby app can effectively help train PCs in supporting and counseling mothers in rural India to exclusively breastfeed. This study contributes to the growing literature demonstrating the applicability of a UCD with an iterative agile development for creating an mHealth app that is most usable for mothers to serve as breastfeeding PCs. The BEST4Baby app was found to be easy to use in support of breastfeeding efforts and provides a framework for its use in future trials.

## Abbreviations

ANM: auxiliary nurse midwife
ASHA: Accredited Social Health Activist
BEST4Baby: Breastfeeding Education Support Tool for Baby
CHW: community health worker
EBF: exclusive breastfeeding
IMR: infant mortality rate
LMIC: low- and middle-income country
mHealth: mobile health
PC: peer counselor
SCT: Social Cognitive Theory
SUS: System Usability Scale
TJU: Thomas Jefferson University
TPB: Theory of Planned Behavior
UCD: user-centered design
